# Nutritional Counseling of Stroke Patients by Neurology Residents, a Call to Action

**DOI:** 10.3389/fneur.2022.940931

**Published:** 2022-07-11

**Authors:** Karima Benameur, Nikhila Gandrakota, Mohammed K. Ali

**Affiliations:** ^1^Department of Neurology, Emory University, Atlanta, GA, United States; ^2^Department of Family and Preventive Medicine, Emory University, Atlanta, GA, United States; ^3^Rollins School of Public Health, Emory University, Atlanta, GA, United States

**Keywords:** stroke, nutrition, residents, counseling, education, diet counseling, post stroke

## Abstract

**Background:**

Poor diet quality has been found to be an independent risk factor for mortality in stroke. However, it is unknown to what extent Neurologists are trained and are comfortable enough to provide dietary counseling to stroke patients.

**Objective:**

To assess the knowledge, attitudes, and practices of neurology residents relating to dietary counseling of stroke patients.

**Methods:**

An online anonymous survey was administered to neurology residents throughout the country between August and November 2019 among a total of 109 (68%) US neurology programs. Self-reported practices and knowledge regarding stroke prevention through nutritional counseling were queried using validated questionnaires.

**Results:**

453 responses out of a potential 672 were received. A minority of residents (12.3%) consistently offered nutritional counseling to stroke patients. 47.7% considered that it was not the neurologist's role to provide nutritional counseling to stroke patients. 83.4% of residents felt that it was the responsibility of the dietician to provide nutritional counseling, yet only 21.4% of residents consistently referred stroke patients to a dietician. 77.9% of respondents felt nutritional counseling is important for stroke patients, yet 65.6% felt they were not adequately trained to provide nutritional counseling.

**Conclusion:**

Neurologists in training believe diet to be an important part of stroke prevention, but practical knowledge and training in nutrition are suboptimal. This study suggests the need to include nutrition as an integral part of neurology training, to ensure neurologists feel empowered to be an important part of the team providing nutritional counseling to stroke patients.

## Introduction

Stroke is the second most common cause of deaths worldwide, after ischemic heart disease, and a leading cause of long-term disability and lost wages. Every year there are ≈795,000 incident strokes in the United States (US) (1). Although the mortality rate from cardiovascular diseases (CVD) had been on the decline for more than a decade, a concerning plateau in that trend and even increase in CVD mortality has emerged recently, largely fueled by the high prevalence of diet-related obesity and diabetes (1).

Poor diet quality has been identified as the leading cause of premature deaths and disability in the United States (2), and has been found to be an independent risk factor for mortality in stroke (3). Diet may be related to stroke through other mechanisms independent of their role in blood pressure and cholesterol (4) such as insulin resistance and inflammation (5). The Primary Prevention of Cardiovascular Disease with a Mediterranean Diet trial (PREDIMED) showed that, in a population with mixed risk factors and treatments, compared to standard low-fat diets, consuming a Mediterranean-style diet was associated with a 30% reduction in major cardiovascular events, mainly driven by less strokes (6). In secondary prevention, the Lyon Diet Heart study (7) documented a 72% reduction of myocardial infarction (MI) and cardiac deaths through dietary modification reflecting the Mediterranean pattern.

Strategies involving physicians to lower cardiovascular risk through diet education and support have been shown to be effective and well received by patients, yet they continue to be under-utilized (8). This may be because very few, an estimated 10–30% of physicians, get adequate training in delivering nutritional guidance and only 8% feel confident in their delivery (9). This data is available for primary care physicians and cardiologists (9, 10); to date, no such data are available for neurologists who deal with CVD risk factors regularly.

The financial burdens of care to patients, families, and healthcare delivery systems from CVD are very high, especially for the uninsured and Medicaid patients (11, 12). Given the potential for CVD event reduction of 30% to 70% from dietary changes (7), the potential for improving public health and lowering healthcare costs by emphasizing nutrition education and practice is substantial.

The American stroke association identifies healthy diets as critical for cardiovascular disease (CVD) risk reduction in general and stroke in particular, and recommends physicians provide dietary guidance for secondary stroke prevention. A recent study on CDC's National Ambulatory Medical Care Survey (NAMCS) data also showed lower rates of diet counseling provided by Neurologists to stroke patients when compared with other physician specialties; however, the factors underlying these low counseling rates are unknown (13). In order to learn more about the knowledge, attitudes, and practices related to nutrition counseling in stroke among Neurologists, a survey was conducted of neurology residents at academic centers across the country.

## Methods

An online survey was developed and adapted from a previously published questionnaire 9. The measurement scales from that instrument had been formally evaluated for reliability and validity and extensively pretested. The survey was adapted to query neurology residents about their nutrition education, as well as knowledge, attitudes, and practice related to nutrition counseling in stroke patients. The study was exempted by our institutional IRB under research exemption 45 cfr 46.101(b)(2).

### Outcomes

The revised, self-administered questionnaire included 35 predominantly closed-ended items utilizing a six-point Likert scale, semantic differential, and true/false questions. The questionnaire was designed to evaluate residents for the following:

1. Knowledge of AHA/ASA Dietary Guidelines (14, 15), Effect of Diet on Cardiovascular Disease, Rationale for Intervention, Appropriate Management Strategies, and Practical Dietary Knowledge.
2. Attitudes Regarding Preparedness to Counsel for Dietary Change, Effectiveness of Dietary Change in Affecting Stroke Risk, Confidence in Counseling Skills, Physician Responsibility for Counseling, Relative Priority of Dietary Counseling, and the Potential Barriers to Counseling, Including Limited Time, Inadequate Educational Materials, and Patient Noncompliance.
3. Demographic Information Such as Level of Training.

The survey was sent to the American Academy of Neurology (AAN) program directors listserv from August to November 2019. A total of 109/158 (68%) US neurology programs were eligible for this survey. Residents were emailed through their program directors with a link to the online survey as well as information on a sweepstakes in which three $50 gift cards would be raffled for incentive. Two additional reminder e-mails were sent to non-respondents over that same time period. Data collection was closed after 8 weeks.

### Analysis

For the purposes of reporting, we dichotomized responses to the six-point Likert scales. Responses 1 through 3 of the scales were classified as disagreement, whereas responses 4 through 6 signified agreement. Residents were classified as in agreement if the mean score on the items of an attitude scale was higher than 3.5.

Residents counseling of their patients' dietary habits in the previous 6 months was based on the AHA/ASA guidelines. Residents' self-reported use of a spectrum of behavior modification strategies for dietary assessment and counseling was measured. Responses were dichotomized at the scale midpoint, and relationships between the dichotomized scales measuring knowledge, attitude, and self-reported behavior were examined.

## Results

A total of 453/672 residents (67.4%) responded to the survey, with an equal breakdown between different levels of training (28.6% PGY2, 27.9% PGY3, 27.3% PGY4) and a minority being interns (9.7%) and fellows (6.5%).

Residents agreed that dietary counseling was a high priority in the care of stroke patients, however they were split about the responsibility about who should deliver that nutritional counseling (Table 1). Almost half (47.7%) seemed to think that it was not the neurologists' responsibility to educate stroke patients about diet and its importance in stroke prevention. The vast majority (83.4%) thought that the responsibility of dietary counseling fell on dieticians, yet only 21.4% referred their patients to a registered dietician.

**Table 1:** Self-reported practice of nutritional counseling of Neurology Residents in the 6 months preceding the survey.

	**Percentage of residents who consistently counseled their patients (*N* = 453)**
During the last 6 months, how often did you discuss diet with your stroke patients During the last 6 months, how often did you discuss diet with your stroke patientsHow often did you discuss diet with your patients	12.3
How often did you determine the amount of fruits and vegetables consumed by your patients	3.5
How often did you determine the amount of fat in your patients diet	6.2
How often did you refer your patients to a dietician	21.4
How often did you inquire about your patient's progress in their dietary changes	10.7
How often did you offer stern warnings about your patients dietary habits	15.9
How often did you set your patient short term dietary goals	5.5

**Figure 1:**
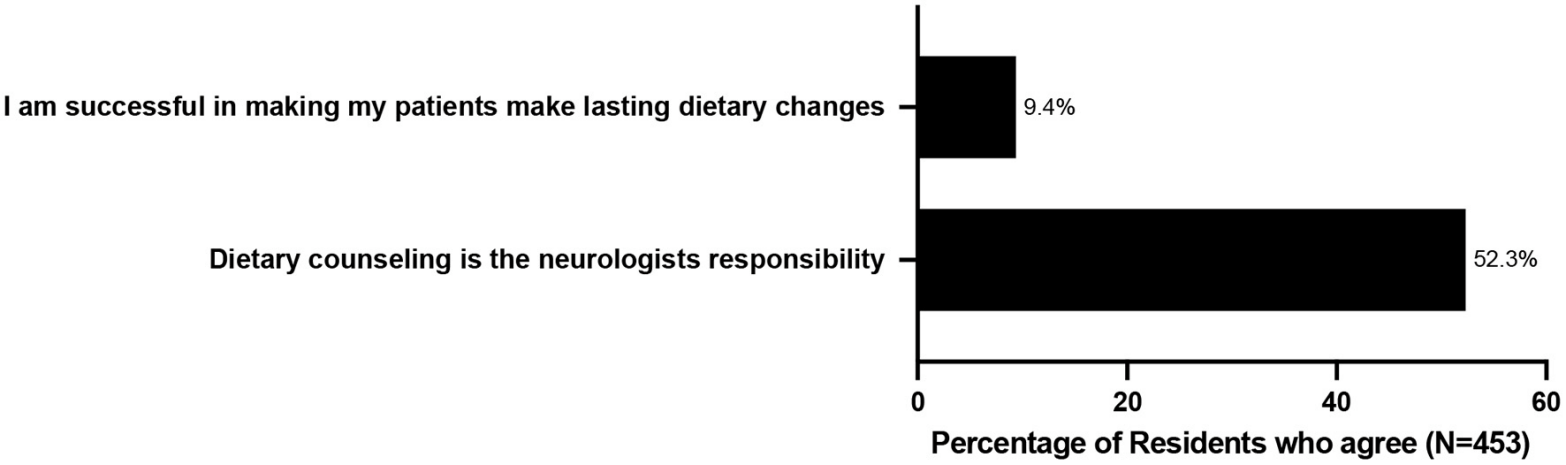
Neurology Residents' Attitudes and Beliefs Regarding Dietary Counseling and Barriers to Counseling.

**Figure 2:**
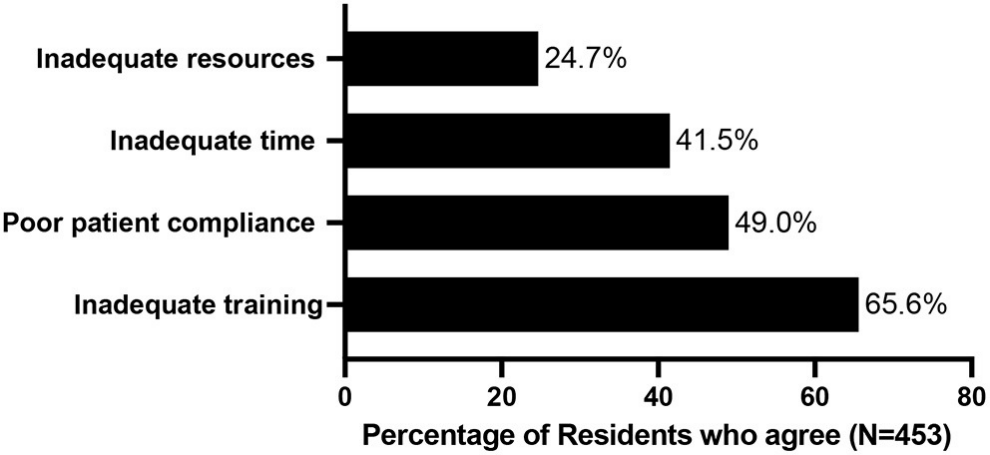
Barriers to Effective Dietary Counseling.

Even though they agreed on the importance of providing dietary counseling, the majority (65.6%) did not feel prepared to do so (Table 1; Figure 1). Even more striking, only 9.4% felt successful or confident in their ability to help patients change their diets. Residents perceived several barriers to educating their patients about nutrition the biggest of which was inadequate training (65.6%), in addition to poor patient compliance (49%), lack of sufficient time (41.5%), and inadequate educational materials (24.7%) (Table 1; Figure 2).

Only 12.3% of residents reported screening for dietary patterns in their patients in the 6 months preceding the survey, and when they did, only 3.5% of residents assessed the amounts of fruits and vegetables their patients consumed on a daily basis, and only 6.3 % assessed the amount of fat in their patients' diet. A very small minority offered their patients well established strategies such as setting up short term dietary goals (5.5%), and inquiring about dietary changes made (10.7%), yet they reported providing stern warnings about dietary habits at times (15.9%) (Table 1).

## Discussion

Our study is the first, to our knowledge, to provide data about nutritional knowledge of and dietary counseling by neurology residents of their stroke patients. Nutritional knowledge and practice has previously been reported in internal medicine residents (9) and cardiology fellows (10), but never in neurology residents. Our data show that although neurology residents agree that nutritional counseling is an important part of stroke care, they do not feel well trained to provide dietary counseling to stroke patients. More alarmingly, our study shows that almost half of them do not feel like it is their responsibility to provide nutritional counseling but rather the dietician, or perhaps the primary care physicians' job, to do that.

The reason for lack of engagement and ownership of neurology residents of nutritional counseling in stroke patients is likely multifactorial, but certainly includes lack of adequate training in nutrition in medical school and neurology residency. To this day, medical schools are only required to provide 25 h of nutrition education throughout the 4 years of education, and even then, a recent survey showed that 71% of medical schools fall short of that requirement (16). After graduating from medical school, nutrition education is nowhere to be found in the neurology curriculum^1^, despite a growing body of evidence showing the impact of diet on neurological diseases in general, and stroke in particular (17). Another reason for the lack of confidence of residents at feeling like they can change their patients' dietary habits lies with training deficiencies at counseling. Indeed, surveys on smoking cessation counseling have shown that knowledge alone is not sufficient to achieve counseling-related behavior changes. In fact, studies have shown a strong association between previous training in smoking cessation counseling and performance of specific counseling activities, but have not shown a link between the simple knowledge of smoking cessation techniques and the performance of counseling activities (18). Essentially, you need knowledge of techniques, as well as training in techniques of smoking cessation to achieve the desired result. Neurology residents don't have the knowledge nor the training in these techniques.

Residents need to be trained in proper methods of providing dietary counseling to stroke patients to be more likely to succeed. These data are consistent with theoretical models of counseling for behavior change. *The 2017 American Heart Association/American Stroke Association Guidelines* (15) emphasize the importance of nutrition and a healthy diet in stroke prevention, and emphasize the importance of first assessing patients' diets, then assessing patients' limitations, and finally encouraging behavior change. In our study, neurology residents fell short of assessing their patients' diets (consumption of fruits and vegetables, content of fat) or in assessing their patients' limitations to adhering to a healthy diet, yet offered stern warnings which could lead to alienating their patients. At a time when stroke education is focused more and more on an interventional approach with simulations and bootcamps of acute strokes (19, 20) and calls for integration of tele-neurology into curriculums of vascular neurology fellowships (21), neurology programs would serve their trainees well by emphasizing and teaching a preventive approach.

### Study Limitations

The response rate to the distributed survey was relatively low. Therefore, it is possible that the results do not accurately reflect the experience and attitudes of all neurology residents. Nevertheless, to our knowledge, this survey is the first and only ever performed among neurologists in training regarding education, attitudes, and practice of nutritional counseling in stroke patients.

## Conclusion

Neurologists in training believe diet to be an important part of stroke prevention, but practical knowledge and training in nutrition are suboptimal. This study is an important first step in shedding a light on this gap. Integration of nutrition and counseling strategies into the curriculum of neurology residency programs may have important benefits for individual patients and population health.

## Data Availability Statement

The raw data supporting the conclusions of this article will be made available by the authors, without undue reservation.

## Ethics Statement

Ethical review and approval was not required for the study on human participants in accordance with the local legislation and institutional requirements. The patients/participants provided their written informed consent to participate in this study.

## Author Contributions

KB and MA contributed to the conception and design of the study. KB contributed to the acquisition and analysis of data. KB, NG, and MA contributed to the drafting the text and preparing the figures and tables. All authors contributed to the article and approved the submitted version.

## Conflict of Interest

The authors declare that the research was conducted in the absence of any commercial or financial relationships that could be construed as a potential conflict of interest.

## Publisher's Note

All claims expressed in this article are solely those of the authors and do not necessarily represent those of their affiliated organizations, or those of the publisher, the editors and the reviewers. Any product that may be evaluated in this article, or claim that may be made by its manufacturer, is not guaranteed or endorsed by the publisher.

## Footnotes

^1^ https://www.acgme.org/globalassets/pfassets/programrequirements/180_neurology_2021.pdf
